# Pornography use, demographic and sexual health characteristics among university students: a gender-based comparative study of non-users, non-problematic users, and problematic users

**DOI:** 10.1186/s12978-024-01841-x

**Published:** 2024-07-10

**Authors:** Zeinab Pouralijan, Beáta Bőthe, Farnaz Farnam

**Affiliations:** 1 Reproductive Health and Midwifery Department, Tehran University of Medical Sciences, Tohid Square, Tehran, Iran; 2 Education Development Center, Babol University of Medical Science, Babol, Iran; 3 Psychology Department, Université de Montréal, Montréal, Canada; 4 Centre de Recherche Interdisciplinaire Sur Les Problèmes Conjugaux Et Les Agressions Sexuelles (CRIPCAS), Université de Montréal, Montréal, Canada

**Keywords:** Pornography, Problematic pornography, Sexual satisfaction, Sexual function, Sexual distress, Iran

## Abstract

**Background:**

Limited gender-based research has compared sexual health among pornography users (PUs) and non-users, including non-problematic pornography users (non-PPUs) and problematic pornography users (PPUs), particularly in non-Western cultures.

**Methods:**

A 2022 cross-sectional study involving 450 Iranian university students categorized participants as PUs or non-users based on 12 months of use. PUs were further classified as non-PPUs or PPUs using the 'Problematic Pornography Use Scale' cutoff point, with comparisons of demographic and sexual variables made between these groups.

**Results:**

Pornography use was reported among 39.6% of students, including 51.7% of men and 33.6% of women. In general, 9.5% of participants were PPUs, including 17.4% of men and 5.6% of women. PUs were mainly men, had fewer children, shorter marriages, lower religiosity, and lower levels of education. Compared with non-users, PUs reported earlier sexual relationships, lower satisfaction with sex frequency and communication, and greater rates of extramarital relationships, masturbation, sexual desire, and sexual distress. PPUs reported more sexual desire, pornography use, masturbation, and extramarital affairs than non-PPUs. Similar patterns in demographics, sexual history, and health were observed in pornography use across genders. The regression indicated being male (OR: 2.42, 95% CI: 1.44–4.06), having lower education (OR: 0.89, 95% CI: 0.81–0.97), fewer children (OR: 0.64, 95% CI: 0.48–0.86), higher masturbation (OR: 1.31, 95% CI: 1.14–1.49), more extramarital relationship (OR: 1.69, 95% CI: 1.07–2.67), less religiosity (OR: 0.87, 95% CI: 0.82–0.93), more sexual excitement (OR: 0.79, 95% CI:0.62–1), and more sexual distress (OR: 1.20, 95% CI: 1.02–1.32) were associated with pornography use.

Two-way ANOVA found no significant effects of gender or pornography use on sexual satisfaction. Women had worse sexual function regardless of usage. Pornography users, regardless of gender, experienced higher sexual distress.

## Background

In the past few decades, with the development of media networks, pornography use has become more widespread [1]. Today, pornography is readily available on personal computers, affordable, and discreet [2]. A national survey in US the in 2014 revealed that 46% of men and 16% of women between the ages of 18 and 39 intentionally viewed pornography in a given week [3]. Also a Chinese study has reported that 94.5% of men and 62% of women view erotic videos online during a 12-month period [4].

Various factors may play a role in pornography use, which can be divided into individual, interpersonal, and social factors. Individual factors include age, gender, religious beliefs [5], marital status, participation in online activities [6], education [7, 8], depression and anxiety [9]. Interpersonal factors of pornography use include family conflicts [10], sexual dissatisfaction [9], and lower levels of marital quality [1]. Regarding social factors, social desirability [5], culture, ethnicity [5, 8] and loneliness [11, 12] can be mentioned. Certain groups are more prone to use pornography [13, 14]. Young people, in particular, are considered an important group in the phenomenon of pornography use due to their increased use of the Internet and social networks, as well as their greater sexual curiosity [15]. Additionally, gender has emerged as one of the most robust indicators of pornography consumption, with a significant majority of users being men, as demonstrated by various studies [3, 7, 16–18]. This gender difference could be explained from an evolutionary perspective that claims that men and women have different short-term and long-term sexual strategies, while men typically exhibit short-term strategies more frequently. These strategies are characterized by a greater willingness to engage in casual sex, lower investment in emotional relationships, and a higher number of sexual partners [19].

Pornography may have various potential effects on people's personal and social lives [20], particularly concerning the sexual health of couples. However, there is no consensus on the relationship between pornography and sexual health [21]. Some studies suggest a positive association between watching pornography and increasing sexual information, learning new sexual techniques, and developing attitudes and awareness toward the opposite sex/gender [22]. On the other hand, a negative association between pornography and sexual health has also been reported [23, 24]. In a meta-analysis in 2017 that measured the association between sexual and relationship satisfaction with pornography, the association was negative and significant [25]. It should be noted that most of the sexual health-related results have been obtained from studies among men and less attention has been given to women.

The potential effects of pornography use on sexuality may vary between non-problematic pornography users (non-PPUs) and problematic pornography users (PPUs). PPUs have difficulty in controlling pornography consumption and reducing usage frequency [26]. It should be considered that problematic pornography is not classified as a distinctive disorder according to the International Classification of Diseases (ICD-11) or the Diagnostic and Statistical Manual of Mental Disorders (DSM-5). However, in ICD-11, this phenomenon is considered as a feature of Compulsive Sexual Behavior Disorder (CSBD) [27]. A few studies that distinguish between problematic and non-problematic pornography usage have shown that sexual function and satisfaction can vary significantly between these two groups. Problematic use may be accompanied by higher levels of sexual avoidance, general sexual compulsivity, and lower sexual satisfaction [28, 29].

The potential effects of watching pornography on one's sex life can relate to sociocultural beliefs, potentially yielding both positive and negative results for sexual health [30]. Some research suggests that religious individuals may encounter more pronounced challenges, such as less sexual satisfaction [31] or more sexual distress due to moral incongruence [32]. However, the majority of studies exploring this phenomenon have been conducted within Western societies, leaving a scarcity of information regarding Asian or Islamic countries.

Iran's university students comprise about 4% of the nation's population [33]. With the ever-increasing use of the Internet and social networks, youth, especially students, are now encountering global sexual scripts [34]. This shift in sexual scripts creates intergenerational conflicts. In this situation, the absence of formal sexual education in school or university curricula, along with the lack of appropriate sexual resources, has led to the emergence of non-official websites on the internet [35] that promote pornography watching. A 2022 national study in Iran, involving 1249 participants found that 27.5% of women and 36% of men reported pornography use in the past year. Moreover, pornography and some related behaviors, such as masturbation or extramarital relationships, are not only unacceptable by society and couples but are also illegal, which may relate to perceived sexual well-being.

To address the above concerns, this article compares sexual satisfaction, function, and distress between university students who use pornography and those who do not, with a focus on gender. In addition, differences in sexual health variables between non-problematic and problematic pornography users were examined. As the present study was exploratory and descriptive in nature, no hypotheses were formed. This paper is part of a comprehensive research project on pornography among university students in Iran.

## Methods

An online controlled cross-sectional study was conducted among Iranian married students between March and November 2022.

### Procedure

The study sample size was determined based on the prevalence of pornography use among married Iranians in 2022. Due to significant gender differences in pornography use in the prevalence observed in the above-mentioned study (40.2% prevalence in men and 27.5% in women), separate gender-based sample sizes were calculated for the current study. University students from all over the country were recruited through convenient online sampling methods. The recruitment involved messages on social media platforms, such as Telegram, WhatsApp, and Instagram, along with advertisements in various groups and locations. A secure web-based platform called “Porsline” hosted the questionnaire, and students received a link to access it. Once completed, the survey data were anonymously transferred to the researchers for analysis. The study began after the research objectives were explained and data confidentiality was ensured. Eligible participants voluntarily provided written informed consent to participate. To prevent bias regarding pornography use, the questionnaire clearly emphasized that the study focused on sexual health issues. As an incentive, participants had the opportunity to win free internet access through a raffle. On average, it took approximately 15 min for participants to complete the survey. After sampling and survey completion, participants were divided into two groups: "pornography non-users" and "users" (PUs) based on a single question about pornography use in the last 12 months. Among pornography users, they were further categorized into non-PPUs and PPUs using the pre-established cutoff point of the Problematic Pornography Use Scale (PPCS) [36].

### Participants

The study included Iranian students who were married for at least six months and lived with their spouse for at least three months within the past six months. The online questionnaire design ensured that only eligible individuals could complete it. Out of 1112 people who opened the survey, 602 people did not meet the study entry criteria. Among the 510 students who met the eligibility criteria, 450 people from all 31 provinces of the country (301 women and 149 men) participated in the study (participation rate = 88.2%). The participants could submit the questionnaire only when they had answered all the items, resulting in no missing data.

### Outcome measures

The survey included demographic and sexuality-related questions, a single-item to identify pornography users and non-users based on their past 12-month frequency of pornography use, and the Problematic Pornography Use Scale (PPCS-6) [36] to distinguish between problematic and non-problematic users. In addition, three standardized scales were used to assess sexual satisfaction, sexual function, and sexual distress as a variables of sexual health.

#### Demographic and sexual history

The demographic characteristics section of the survey included six items: age, marriage duration, education level (counting from the first grade of elementary school), number of children, economic status, and religiosity. The latter variable was assessed using three questions based on previous work [37], with scores ranging from 3 to 18, where higher scores indicated greater religiosity. The section on sexual history comprised six items: age at first sexual intercourse, frequency of sexual activity during the last three months, frequency of masturbation in the last month, sexual communication, satisfaction with sexual frequency, and any involvement in extramarital relationships after marriage.

#### Pornography use

To clarify the meaning of "pornography use," the researchers provided participants with a definition of pornography. "Pornography refers to the use of any photo, video, text, etc., which is prepared solely for the purpose of creating sexual thoughts and stimulation, and shows sexual activities such as vaginal, oral, and anal intercourse in detail, depicting male and female reproductive organs." To differentiate pornography content from romantic scenes, a note was added: "Watching a movie containing romantic relationship scenes, such as kissing or hugging, is not considered pornography." Based on participants' responses to a single question asking whether they had used pornography in the past 12 months, with the possible responses being "yes" or "no," they were categorized into pornography non-users and pornography users. The frequency of PU was assessed using a single-item question: 'How often do you watch pornography?' with daily, weekly, monthly, and yearly response options.

#### Problematic pornography consumption scale (PPCS-6)

In the subsequent step, among PUs, problematic use was assessed with the PPCS-6 [36]. This scale is the shortened version of the 18-item tool and examines symptoms of PPU experienced within the past six months. Participants rated each of the six items on a seven-point Likert scale, ranging from 1 (never) to 7 (always), such as “I neglected other leisure activities as a result of watching porn.” The total score ranged from 6 to 42, with a pre-stablished cut-off point of ≥ 20, suggesting potential problematic use. The Persian version of the PPCS scale demonstrated good validity and reliability, as evidenced by a Cronbach's alpha (α) of 0.97 and an intra-class correlation coefficient (ICC) of 0.97 [38].

#### Global measure of sexual satisfaction (GMSEX)

Sexual satisfaction was evaluated using the five-item GMSEX, which employs a seven-point semantic difference scale (in general, how would you describe your satisfaction with sex with your partner? (Very unpleasant = 1; Very pleasant = 7). The total score on this scale ranges from 5 to 35, with higher scores indicating greater satisfaction [39]. The Persian version of the GMSEX scale demonstrated good reliability, with a Cronbach's alpha of 0.88 and an ICC ranging from 0.70 to 0.95 (at the 95% CI) [40].

#### Arizona sexual experience scale

This study used the five-item Arizona Sexual Experience Scale to assess sexual function problems in both women and men. This scale measures sexual desire, sexual excitement, erection or lubrication, the ability to reach orgasm, and orgasm satisfaction through five items such as “How easily can you reach an orgasm?” Participants responded to the items using a six-point Likert scale, with options ranging from 1 (extremely easily) to 6 (never each). The total score on the scale ranged between 5 and 30, with higher scores in each dimension indicating a greater degree of sexual problems and a score above 18 indicating sexual dysfunction. The Arizona Sexual Experience Scale has demonstrated adequate validity and reliability in previous studies [41], including in the Iranian population [42]. Additionally, the study assessed Dyspareunia in female participants by asking a single question: "Do you have pain during intercourse?" Participants responded using a five-point Likert scale, with options ranging from 1 (never) to 5 (always). The total score of this question ranged from 1 to 5, with higher scores indicating greater sexual pain. Cronbach’s alpha analysis indicated that the Arizona demonstrated excellent internal consistency (Cronbach’s alpha = 0.91) [41].

#### The short sexual distress scale (SDS-3)

Sexual distress measured by the SDS-3 in men and women. Items were scored on a five-point Likert scale, ranging from 0 (never) to 4 (always), such as “Have you been distressed about your sex life?” The total score ranged between 0 and 12 points, and a higher score indicated greater sexual distress. The validity and reliability of this scale have been examined in previous studies [43], including in the Iranian population [44]. Cronbach's alpha coefficient was 0.89 for women and 0.87 for men [45].

### Statistical analyses

The comparisons between the aforementioned groups of pornography users and non-users, as well as between problematic and non-problematic users in the study were conducted using either independent t-tests or Mann–Whitney U-tests, depending on whether the assumptions of the parametric tests were met. For categorical variables, results are presented as N (percentages) and were compared using either the chi-square test or Fisher’s exact test. Two-way ANOVA was used to examine the effects of two factors, gender (two levels), and pornography use (three levels: Non-users, PUs, and PPUs), and their interaction on three main variables of sexual health, including sexual satisfaction, sexual function, and sexual distress. A Bonferroni adjusted test was conducted to decrease type 1 error, and the significance level was considered to be 0.01. Cohen’s *d*s of 0.2, 0.5, and 0.8 are considered as small, medium, and large effect sizes, respectively. The backward logistic method was employed for the regression analysis. The backward method is chosen to eliminate redundant or potentially collinear variables and enhance the interpretability of the mode. In the variable selection process, all those with a significance level of 0.2 [46] were inserted into the model to predict pornography use. All the statistical analyses were performed using SPSS version 25.

### Ethics

The study adhered to scientific and ethical standards of the Declaration of Helsinki and was approved by the ethics committee of Tehran University of Medical Sciences, with the ethical code IR.TUMS.FNM.REC.1400.179.

## Results

Among the 450 participants, 301 were women and 149 were men. Of the total participants, 272 individuals (60.4%) did not use pornography, comprising 72 men (26.5%) and 200 women (73.5%). 178 students (39.6%) disclosed using pornography, with a prevalence rate of 33.6% among women and 51.7% among men. According to the PPCS-6 scale cut-off, 24.2% of pornography users or 9.5% of the overall population were classified as PPUs. Within the group of female pornography users, 16.8% reported problematic usage (equivalent to 5.6% of the total population). Among male pornography users, 33.8% acknowledged problematic usage (representing 17.4% of the general population).

### Demographic and sexual history of pornography users and non-users

Compared to non-users, PUs were more likely to be men (*P* < 0.001), 3 years younger (*P* < 0.001), and had fewer years of education (*P* = 0.004). PUs also had lower religiosity scores (*P* < 0.001), shorter marriage durations (*P* < 0.001), and fewer children (*P* < 0.001). In terms of sexual behaviors, PUs reported significantly less sexual communication (*P* = 0.008), a lower age at first sexual experience (*P* < 0.001), a greater frequency of masturbation (*P* < 0.001), and a greater occurrence of extramarital relationships (*P* < 0.001). While there were no significant differences between the two groups in terms of sex frequency during the past three months, PUs expressed lower satisfaction with their sex frequency (*P* = 0.002). The pattern of differences in demographic and sexual parameters between female PUs and non-users was consistent with that of the general population. The duration of marriage and the number of children were significantly lower for women than men. Among all variables, religiosity had the greatest effect size (d = 0.6), especially between male users and non-users (d = 0.7) (Tables 1, 2).

**Table 1:** Gender differences between studied groups

Groups	Male	Female	*p*-value £
N (%)		N (%)	
Non-PUs	72 (26.5%)	200 (73.5%)	0.0001
PUs	77 (43.3%)	101 (56.7%)	
Non-PPUs	51 (37.8%)	84 (62.2%)	0.009
PPUs	26 (60.5%)	17 (39.5%)	

*N* Number, % Percentage, *PUs* Pornography users, *Non-PUs* Non pornography users, *PPUs* Problematic pornography users, *Non-PPUs* Non-problematic pornography users, £ Based on the Chi-square test

**Table 2:** Demographic and sexual characteristics of pornography users and non-users

Variable	Women (*N* = 301)		*P*-value	Cohen	Men (*N* = 149)		*P*-value	Cohen	All (*N* = 450)		*P*-value	Cohen d
	**PUs**	**Non-PUs**		**d**	**PUs**	**Non-PUs**		**d**	**PUs**	**Non-PUs**		
	**N (%)**	**N (%)**			**N (%)**	**N (%)**			**N (%)**	**N (%)**		
	101 (33.6%)	200 (66.4%)			77 (51.7%)	72 (48.3%)			178 (39.6%)	272 (60.4%)		
	**M (SD)**	**M (SD)**	**ǂ**		**M (SD)**	**M (SD)**	**ǂ**		**M (SD)**	**M (SD)**	**ǂ**	
**Age**	26.83 ± 5.37	30.39 ± 8.05	**0.0001**	0.6	31.00 ± 7.54	34.26 ± 6.61	**0.006**	0.4	28.63 ± 6.71	31.41 ± 7.87	**0.0001**	0.4
**Marriage duration (year)**	3.95 ± 3.87	6.78 ± 7.03	**0.0001**	0.5	5.09 ± 5.85	6.99 ± 6.18	0.055	0.3	4.44 ± 4.85	6.84 ± 6.81	**0.0001**	0.4
**Education (year)**	16.44 ± 2.51	17.37 ± 2.94	**0.007**	0.3	17.63 ± 20.03	18.72 ± 2.81	**0.008**	0.1	16.96 ± 2.38	17.73 ± 2.97	**0.004**	0.3
**Religiosity**	8.47 ± 3.99	10.62 ± 3.79	**0.0001**	0.5	9.03 ± 4.05	11.66 ± 3.35	**0.0001**	**0.7**	8.71 ± 4.02	10.90 ± 3.70	**0.0001**	0.6
**Age at first sex**	21.30 ± 4.13	22.62 ± 4.43	**0.013**	0.3	22.04 ± 4.55	24.76 ± 4.79	**0.001**	0.6	21.62 ± 4.32	23.29 ± 4.50	**0.0001**	0.4
**Sex frequency (3 months)**	22.53 ± 21.91	20.72 ± 19.31	0.463	0.1	21.30 ± 17.73	21.60 ± 18.82	0.922	0	22.01 ± 20.17	20.95 ± 19.15	0.578	0
**Masturbation (1 month)**	3.33 ± 7.92	0.42 ± 1.89	**0.0001**	0.5	6.28 ± 16.59	0.25 ± 0.96	**0.002**	0.5	4.59 ± 12.45	0.38 ± 1.69	**0.0001**	0.5
	**(%)**	**(%)**	**£**		**(%)**	**(%)**	**£**		**(%)**	**(%)**	**£**	
**Children**												
0	74.30%	53.00%	**0.0001**		62.30%	43.10%	0.021		69.10%	50.40%	**0.0001**	
1	21.80%	25.50%			18.20%	22.20%			20.20%	24.60%		
2	3.00%	16.00%			18.20%	25.00%			9.60%	18.40%		
3	1.00%	5.00%			0.00%	8.30%			0.60%	5.90%		
4	0.00%	0.50%			1.30%	1.40%			0.60%	0.70%		
**Economic**												
Unpleasant	13.90%	11.00%	0.734		23.40%	18.10%	0.726		18.00%	12.90%	0.305	
Medium	71.30%	75.00%			61.00%	65.30%			66.90%	72.40%		
Pleasant	14.90%	14.00%			15.60%	16.70%			15.20%	14.70%		
**Sex communication**												
Very easy	35.60%	51.00%	0.034		44.20%	63.90%	0.091		39.30%	54.40%	**0.008**	
Easy	34.70%	25.00%			26.00%	18.10%			30.90%	23.20%		
Hard	24.80%	16.50%			22.10%	11.10%			23.60%	15.10%		
Very hard	5.00%	7.50%			7.80%	6.90%			6.20%	7.40%		
**Sex frequency satisfaction**												
Less than desired	39.60%	26.00%	0.046		54.50%	40.30%	0.121		46.10%	29.80%	**0.002**	
Desired	55.40%	66.00%			42.90%	58.30%			50.00%	64.00%		
More than desired	5.00%	8.00%			2.60%	1.40%			3.90%	6.30%		
**Extramarital relationship**												
I don’t want to answer	5.00%	4.00%	**0.0001**		5.20%	4.20%	**0.009**		5.10%	4.00%	**0.0001**	
No	83.20%	94.50%			72.70%	90.30%			78.70%	93.40%		
Yes	11.90%	1.50%			22.10%	5.60%			16.30%	2.60%		

*M* Mean, *SD* Standard deviation, % Percentage, *PUs* Pornography Users, *Non-PUs* non-Pornography Users, **ǂ**: Based on Independent t-tests, £: Based on Chi-square tests

### Sexual health variables across pornography users and non-users

Sexual satisfaction was not significantly different between PUs and non-users, although it was lower for PUs. General sexual function also indicated no significant difference, although it was slightly better for PUs. However, PUs reported higher levels of sexual desire (*P* = 0.001). Dyspareunia, measured only among women, was higher in PUs, nearing statistical significance (*P* = 0.01). The only sexual health variable that showed meaningful differences between these two groups was sexual distress (*P* = 0.001). The largest effect size was related to sexual distress (d = 0.5), especially among male users and non-users (d = 0.7) (Table 3).

**Table 3:** Sexual health variables across pornography users and non-users

Variable	Women (*N* = 301)		*P*-value	Cohen d	Men (*N* = 149)		*P*-value	Cohen d	All (*N* = 450)		*P*-value	Cohen d
	**PUs**	**Non-PUs**			**PUs**	**Non-PUs**			**PUs**	**Non-PUs**		
	N (%)	N (%)			N (%)	N (%)			N (%)	N (%)		
	101 (33.6%)	200 (66.4%)			77 (51.7%)	72 (48.3%)			178 (39.6%)	272 (60.4%)		
	**M (SD)**	**M (SD)**	**ǂ**		**M (SD)**	**M (SD)**	**ǂ**		**M (SD)**	**M (SD)**	**ǂ**	
**Sexual satisfaction**	26.79% ± 7.60	28.14 ± 6.96	0.126	0.2	27.12 ± 6.24	28.29 ± 6.14	0.255	0.2	26.93 ± 7.03	28.18 ± 6.74	0.061	0.2
**Sexual distress**	3.49 ± 3.00	2.33 ± 2.61	**0.001**	0.4	4.00 ± 2.60	2.26 ± 2.75	**0.0001**	**0.7**	3.71 ± 2.83	2.31 ± 2.64	**0.0001**	0.5
**Sexual function**^**a**^	13.42 ± 4.21	13.54 ± 4.93	0.843	0	11.06 ± 3.57	11.22 ± 4.13	0.804	0.1	12.40 ± 4.11	12.92 ± 4.84	0.236	0.1
Desire	2.43 ± 1.15	2.76 ± 1.04	0.012	0.3	2.14 ± 0.94	2.35 ± 0.93	0.187	0.2	2.30 ± 1.07	2.65 ± 1.03	**0.001**	0.3
Excitement	2.64 ± 1.00	2.80 ± 1.15	0.248	0.1	2.23 ± 0.85	2.33 ± 0.97	0.509	0.1	2.47 ± 0.96	2.68 ± 1.12	0.042	0.2
Lubrication or erection	2.60 ± 1.15	2.46 ± 1.21	0.306	0.1	2.23 ± 0.88	2.25 ± 1.05	0.919	0	_	_	_	
Orgasm	3.11 ± 1.28	2.98 ± 1.36	0.414	0.1	2.05 ± 0.99	2.07 ± 0.93	0.913	0	2.65 ± 1.28	2.74 ± 1.32	0.508	0.1
Orgasm satisfaction	2.64 ± 1.20	2.55 ± 1.31	0.548	0.1	2.40 ± 1.06	2.22 ± 1.03	0.298	0.2	2.54 ± 1.15	2.46 ± 1.25	0.515	0.1
	**(%)**	**(%)**	**£**		**(%)**	**(%)**			**(%)**	**(%)**	**£**	
**Dyspareunia**												
Never	20.80%	27.50%	0.019		_	_	_		20.80%	27.50%	0.019	
Less than 25% of times	40.60%	51.50%							40.60%	51.50%		
About half of times	23.80%	12.50%							23.80%	12.50%		
More than 75% of times	7.90%	6.00%							7.90%	6.00%		
Always	6.90%	2.50%							6.90%	2.50%		

*M* Mean, *SD* Standard deviation, % Percentage, *PUs* Pornography Users, *non-Us* non-Pornography Users, **ǂ**: Based on Independent t-tests, £: Based on Chi-square tests, ^a^The higher score in this variable and its dimensions imply higher levels of sexual dysfunction

### Demographic and sexual history across non-PPUs and PPUs

Of the 178 PUs, 75.8% (*n* = 135) were non-PPUs, with 37.8% being men (*n* = 51) and 62.2% being women (*n* = 84). In this survey, 24.2% (*n* = 43) were classified as potentially PPUs, with 60.5% being men (*n* = 26) and 39.5% being women (*n* = 17), indicating a significantly greater frequency among men (*P* = 0.009) (Table 1). PPUs reported more frequent pornography use (*P* < 0.001), masturbation (*P* = 0.005), and extramarital affair (*P* = 0.004) than non-PPUs. The greatest effect size was related to masturbation (d = 0.6), especially between women users and non-users (d = 0.9) (Table 4).

**Table 4:** Demographic and sexual information of problematic and non-problematic pornography users

**Variable**	PUs women (*N* = 101)		***P*-value**	**Cohen d**	PUs men (*N* = 77)		***P*-value**	**Cohen d**	All PUs (*N* = 178)		***P*-value**	**Cohen d**
	**PPUs**	**Non-PPUs**			**PPUs**	**Non-PPUs**			**PPUs**	**Non-PPUs**		
	**N (%)**	**N (%)**			**N (%)**	**N (%)**			**N (%)**	**N (%)**		
	17 (16.8%)	84 (83.2%)			26 (33.8%)	51 (66.2%)			43 (24.2%)	135 (75.8%)		
	**M (SD)**	**M (SD)**	**ǂ**		**M (SD)**	**M (SD)**	**ǂ**		**M (SD)**	**M (SD)**	**ǂ**	
**Problematic use score**	25.88 ± 5.87	10.39 ± 4.04	0.0001	3.1	25.34 ± 5.59	11.88 ± 4.32	0.0001	2.7	25.55 ± 5.64	1.95 ± 4.19	0.0001	**4.7**
**Age**	25.52 ± 3.95	27.09 ± 5.59	0.275	0.3	30.65 ± 8.54	31.17 ± 7.07	0.776	0.1	28.62 ± 7.47	28.63 ± 6.48	0.994	**0**
**Marriage duration (year)**	2.47 ± 1.77	4.25 ± 4.11	**0.006**	0.6	4.57 ± 5.62	5.35 ± 6.01	0.586	0.1	3.74 ± 4.59	4.67 ± 4.92	0.277	**0.2**
**Education (year)**	16.41 ± 2.59	16.45 ± 2.50	0.952	0	17.65 ± 2.13	17.62 ± 1.99	0.975	0	17.16 ± 2.37	16.89 ± 2.39	0.525	**0.1**
**Religious**	8.41 ± 4.15	8.48 ± 3.98	0.943	0	9.00 ± 3.92	9.05 ± 4.15	0.952	0	8.76 ± 3.98	8.70 ± 4.04	0.928	**0**
**Age at first sex**	19.41 ± 3.44	21.68 ± 4.17	0.039	0.6	21.88 ± 4.14	22.57 ± 4.45	0.516	0.5	20.91 ± 4.02	21.96 ± 117.83	0.54	**0.3**
**Sex frequency (3 months)**	24.35 ± 21.59	22.17 ± 22.08	0.71	0.1	27.08 ± 21.33	18.47 ± 15.10	0.046	0.5	25.98 ± 21.22	20.77 ± 19.76	0.145	**0.3**
**Masturbation (1 month)**	12.29 ± 16.01	1.51 ± 2.53	0.014	**0.9**	10.60 ± 20.33	4.16 ± 14.16	0.112	0.4	11.29 ± 18.51	2.51 ± 8.97	**0.005**	**0.6**
	**(%)**	**(%)**	**£**		**(%)**	**(%)**	**£**		**(%)**	**(%)**	**£**	
**Porn frequency**												
Daily	5.90%	1.20%	**0.0001**		46.20%	5.90%	**0.0001**		30.20%	3.00%	**0.0001**	
Weekly	41.20%	13.10%			30.80%	21.60%			34.90%	16.30%		
Monthly	52.90%	38.10%			23.10%	35.30%			34.90%	37.00%		
Yearly	0.00%	47.60%			0.00%	37.30%			0.00%	43.70%		
**Children**												
0	94.10%	70.20%	0.288		65.40%	60.80%	0.961		76.70%	66.70%	0.462	
1	5.90%	25.00%			15.40%	19.60%			11.60%	23.00%		
2	-	3.60%			19.20%	17.60%			11.60%	8.90%		
≥ 3	-	1.20%			-	2.00%			-	1.40%		
**Economic**												
Unpleasant	23.50%	11.9&	0.323		23.10%	23.50%	0.134		23.30%	16.30%	0.092	
Medium	58.80%	73.80%			50.00%	66.70%			53.50%	71.10%		
Pleasant	17.60%	14.30%			26.90%	9.80%			23.30%	12.60%		
**Sex communication**												
Very easy	29.40%	36.90%	0.312		38.50%	47.10%	0.055		34.90%	40.70%	0.033	
Easy	23.50%	36.90%			19.20%	29.40%			20.90%	34.10%		
Hard	41.20%	21.40%			23.10%	21.60%			30.20%	21.50%		
Very hard	5.90%	4.80%			19.20%	2.00%			14.00%	3.70%		
**Sex frequency satisfaction**												
Less than desired	58.80%	35.70%	0.204		57.70%	52.90%	0.749		58.10%	42.20%	0.182	
Desired	41.20%	58.30%			38.50%	45.10%			39.50%	53.30%		
More than desired	-	6.00%			3.80%	2.00%			2.30%	4.40%		
**Extramarital relationship**												
I don’t want to answer	-	6.00%	**0.001**		7.70%	3.90%	0.563		4.70%	5.20%	**0.004**	
No	58.80%	88.10%			65.40%	76.50%			62.80%	83.70%		
Yes	41.20%	6.00%			26.90%	19.60%			32.60%	11.10%		

*M* Mean, *SD* Standard deviation, % Percentage, *PPUs* Problematic Pornography Users, *non-PPUs* non- Problematic Pornography Users, **ǂ**: Based on Independent t-tests, £: Based on Chi-square tests

### Sexual health variables across non-PPUs and PPUs

Sexual satisfaction showed no meaningful differences between PPUs and non-PPUs, although it was lower among PPUs. There was no significant difference in general sexual function, although it was more unfavorable in PPUs. However, for the sexual function components, sexual desire was greater among PPUs (*P* = 0.008). Sexual distress was meaningfully higher among PPUs than among non-PPUs (*P* = 0.001). The largest effect size was related to sexual distress (d = 0.7), especially among men users’ vs non-users (d = 0.9) (Table 5).

**Table 5:** Sexual health variables of problematic and non-problematic pornography users

Variable	PUs Women (*N* = 101)		*P*-value	Cohen d	PUs Men (*N* = 77)		*P*-value	Cohen d	All PUs (*N* = 178)		*P*-value	Cohen d
	**PPUs**	**Non-PPUs**			**PPUs**	**Non-PPUs**			**PPUs**	**Non-PPUs**		
	N (%)	N (%)			N (%)	N (%)			N (%)	N (%)		
	17 (16.8%)	84 (83.2%)			26 (33.8%)	51 (66.2%)			43 (24.2%)	135 (75.8%)		
	**M (SD)**	**M (SD)**	**ǂ**		**M (SD)**	**M (SD)**	**ǂ**		**M (SD)**	**M (SD)**	**ǂ**	
**Sexual satisfaction**	25.47 ± 6.47	27.05 ± 7.82	0.435	0.2	26.84 ± 7.60	27.27 ± 5.51	0.8	0.1	26.30 ± 7.13	27.14 ± 7.01	0.498	**0.1**
**Sexual distress**	5.64 ± 2.71	3.05 ± 2.88	**0.001**	**0.9**	4.80 ± 3.09	3.58 ± 2.22	0.051	0.5	5.13 ± 2.94	3.25 ± 2.65	0.0001	0.7
Sexual function*	14.00 ± 3.39	13.30 ± 4.37	0.541	0.2	11.00 ± 4.25	11.09 ± 3.22	0.91	0	12.18 ± 4.16	12.47 ± 4.10	0.69	**0.1**
Desire	2.00 ± 0.93	2.51 ± 1.17	0.095	0.5	1.88 ± 0.90	2.27 ± 0.94	0.086	0.4	1.93 ± 0.91	2.42 ± 1.09	**0.008**	**0.5**
Excitement	2.41 ± 1.06	2.69 ± 0.99	0.3	0.3	2.19 ± 0.93	2.25 ± 0.82	0.764	0.1	2.28 ± 0.98	2.53 ± 0.95	0.144	**0.3**
Lubrication or erection	2.94 ± 1.56	2.54 ± 1.04	0.186	0.3	2.23 ± 0.95	2.24 ± 0.86	0.983	0	_	_	_	
Orgasm	3.76 ± 1.14	2.98 ± 1.28	0.021	0.6	2.12 ± 1.14	2.02 ± 0.92	0.693	0.1	2.77 ± 1.39	2.61 ± 1.24	0.498	**0.1**
Orgasm satisfaction	2.88 ± 1.11	2.60 ± 1.22	0.373	0.2	2.58 ± 1.32	2.31 ± 0.90	0.371	0.2	2.70 ± 1.24	2.49 ± 1.11	0.301	**0.2**
	**(%)**	**(%)**	**£**		**(%)**	**(%)**			**(%)**	**(%)**	**£**	
**Dyspareunia**												
Never	5.90%	23.80%	0.478		_	_	_		5.90%	23.80%	0.478	
Less than 25% of times	52.90%	38.10%							52.90%	38.10%		
About half of times	29.40%	22.60%							29.40%	22.60%		
More than 75% of times	5.90%	8.30%							5.90%	8.30%		
Always	5.90%	7.10%							5.90%	7.10%		

*M* Mean, *SD* Standard deviation, % Percentage, *PPUs* Problematic Pornography Users, *non-PPUs* non-Problematic Pornography Users, **ǂ** Based on Independent t-tests, £ Based on Chi-square tests, *The higher score in this variable and its dimensions imply higher levels of sexual dysfunction

### Two-way ANOVAs

Three distinctive two-way ANOVAs were conducted for the main sexual health variables including sexual satisfaction, function, and distress (Table 6). In each ANOVA, the effects of gender (male, female), pornography use (pornography users, non-users) and their interaction were examined. The results showed no significant main effect for gender [*F* (1, 444) = 0.001, *P* = 0.511], pornography [*F* (2, 444) = 0.009, *P* = 0.138], or interaction between gender and pornography [*F* (2, 444) = 0.001, *P* = 0.869] on sexual satisfaction. The significant main effect for gender [*F* (1, 444) = 0.042, *P* = 0.000], but not for pornography [ *F* (2, 444) = 0.000, *P* = 0.910], or the interaction between gender and pornography [*F* (2, 444) = 0.001, *P* = 0.882] was observed for sexual function and women reported lower levels of function than men. The results indicated no significant main effect for gender [*F* (1, 444) = 0.000, *P* = 0.711] or interaction between gender and pornography [*F* (2, 444) = 0.005, *P* = 0.326] on sexual distress. However, pornography had a significant effect [ F (2, 444) = 0.091, *P* = 0.000] on sexual distress, as sexual distress in PUs compared to non-users (*P* = 0.003), and in PPUs compared to PUs (*P* = 0.000) significantly raised.

**Table 6:** Between-Subjects Effects for two gender and pornography use factors on sexual health variables

Source	Type III Sum of Squares	df	Mean Square	F	Sig.	Partial Eta Squared
**Dependent Variables: Sexual Satisfaction**						
Pornography	188.871	2	94.436	1.991	.138	.009
Gender	20.552	1	20.552	.433	.511	.001
Pornography*Gender	13.283	2	6.641	.140	.869	.001
a. R Squared = .010 (Adjusted R Squared = -.001)						
**Dependent Variables: Sexual Function**						
Pornography	3.755	2	1.878	.095	.910	.000
Gender	383.852	1	383.852	19.348	**.000**	.042
Pornography*Gender	5.003	2	2.502	.126	.882	.001
a. R Squared = .060 (Adjusted R Squared = .050)						
**Dependent Variables: Sexual Distress**						
Pornography	320.585	2	160.292	22.272	**.000**	.091
Gender	.987	1	.987	.137	.711	.000
Pornography*Gender	16.178	2	8.089	1.124	.326	.005
a. R Squared = .097 (Adjusted R Squared = .086)						

### Logistic regression

The logistic regression analysis indicated significant associations between several variables and pornography use (Table 7). Being male (OR: 2.42, 95% CI: 1.44–4.06), having lower education (OR: 0.89, 95% CI: 0.81–0.97), less children (OR: 0.64, 95% CI: 0.48–0.86), higher masturbation (OR: 1.31, 95% CI: 1.14–1.49), more extramarital relationship (OR: 1.69, 95% CI: 1.07–2.67), less religiosity (OR: 0.87, 95% CI: 0.82–0.93), more sexual excitement (OR: 0.79, 95% CI:0.62–1), and more sexual distress (OR: 1.20, 95% CI: 1.02–1.32) were associated with higher pornography use.

**Table 7:** Predictors of pornography usage by logistic regression

Variables in the Equation				
	Sig.	Exp (B)	Lower	Upper
Gender	.001	2.424	1.446	4.062
Education	.012	.892	.816	.975
Children	.003	.649	.486	.866
Masturbation (monthly)	.000	1.310	1.147	1.497
Sexual excitement	.058	.797	.629	1.008
Extramarital relationship	.023	1.695	1.074	2.675
Religiosity	.000	.875	.823	.931
Sexual distress	.000	1.209	1.102	1.325
Constant	.082	4.802		

Variable(s) entered on step 1: Age, Gender, Education, Marriage duration (year), Children, Age at first sex

Sex frequency satisfaction, Masturbation, Sex communication, Sexual desire, Sexual excitement, Extramarital relationship, Religiosity, Sexual satisfaction, and Sexual distress

## Discussion

The present study is one of the few studies that compare demographic characteristics, sexual history, and sexual well-being among pornography users and non-user Iranian students, as well as between PPU and non-PPU based on gender**.**

The findings of the present study showed that 51% of male and 33% of female students used pornography in the past year in the present Iranian sample, in line with previous studies’ findings suggesting a male dominance among pornography users [3, 7, 17], and among college students [47].

Concerning demographic characteristics, significant differences were observed between non-users and PUs in all assessed variables, except for economic conditions. Consistent with previous studies [7, 8], younger students tended to watch more pornography, likely because of their increased access to the Internet and digital devices [13]. Similarly, those with shorter marriages also shared a similar inclination, possibly influenced by curiosity and heightened sexual excitement [48] or gaining sexual knowledge. A potential explanation for the reduced use of pornography among older individuals, especially older males, could be attributed to the diminishing influence of marriage and fatherhood on basal testosterone levels [49]. These findings highlighted the need for implementing suitable education in universities and introducing reliable sources of sexual information as reasonable strategies for policymakers. According to prior surveys [42], individuals with less education and fewer children [40] were more likely to use pornography, probably due to having more free time. A potential explanation for the reduced use of pornography among older individuals, especially older males, could be attributed to the diminishing influence of marriage and fatherhood on basal testosterone levels. Furthermore, the mean religiosity score was lower for pornography users. In fact, the religiosity difference between PUs and non-PUs showed a large effect size, especially for men (d = 0.7). Other studies also suggest that religion might have acted as a deterrent to watch pornographic content, likely due to increased feelings of guilt or shame [30, 47]. However, distinctions in demographic traits between PPUs and non-PPUs were not prominent.

As for sexual history, all variables differed between users and non-users, except for sex frequency. The age at first sexual activity among pornography users was lower (d = 0.4), which is similar to the findings of other studies [50, 51]. PUs have significantly greater rates of masturbation (5 vs. 0.4 times a month for non-users), extramarital relationships (16% vs. 2% for non-users), and less sex communication with spouses [52]. Contrary to some surveys suggesting that pornography facilitates sexual communication [53, 54], this study, along with surveys conducted in religious contexts [55, 56] showed that PUs might have experienced difficulties in sexual conversation with their spouse. Consistent with the result of a previous study [43], the findings of this study indicated that PUs expressed dissatisfaction with their frequency of sexual activity. Surprisingly, this feeling persisted despite an equal frequency of sexual intercourse between PUs and non-users (22 vs. 21 sexual intercourses in non-users during the past three months). Accordingly, clinicians can play a role in preventing potential adverse consequences of pornography use through pornography literacy programs by promoting sexual communication and enabling young couples to design their sexual lives based on their preferences.

Our results alongside many other studies stated [57, 58] implied that PPUs reported higher rates of monthly masturbation (11.2 vs 2.5 times in non-PPUs), sex frequency (30.2% vs 3% daily use), and extramarital relationships (32.6% vs 11.1%) than non-PPUs. Masturbation showed the largest effect sizes among PPUs and non-PPUs. This result corroborates previous findings suggesting that these behaviors may co-occur [57, 58]. Interestingly, religiosity was not significantly different between problematic and non-problematic users. The score for problematic use was significantly different (25.5 in PPUs vs 1.9 in non-PPUs), while the religiosity scores were the same (8.7 vs 8.7). The observed meaningful higher sexual desire, masturbation, pornography frequency, and sexual affairs in PPUs; and equal religiosity suggest that the higher scores on the problematic pornography scale in PPUs cannot be explained solely by differences in moral incongruence [30], even in a religious context and support the notion that PPU may be a distinct problem.

The trend of sexual satisfaction decreased from non-users to PPUs. However, two-way ANOVA revealed that neither gender nor pornography was associated sexual satisfaction. Nevertheless, the small sample size, particularly in the PPU group (43 people), hinders the ability to draw reliable conclusions. It would be beneficial to conduct future studies with larger sample sizes within the PPUs group.

There were no significant differences in general sexual function between the groups. However, findings revealed that sexual function was solely related to gender, and women reported poorer function than men did, regardless of pornography use. In fact, in all five dimensions of sexual function, women reported lower scores. One notable finding was a significant increase in sexual desire from non-users to users (d = 0.3), and further from non-PPUs to PPUs (d = 0.5) in this survey. The greater amount of dyspareunia observed in this survey, a less frequently assessed finding in other studies, may be due to engaging in specific sexual behaviors such as anal intercourse [59]. However, due to the cross-sectional nature of this study, it is also possible that those with greater sexual pain are less likely to have sex and turn to pornography for pleasure.

Sexual distress significantly increased from non-users to PPUs. In fact, sexual distress showed one of the most significant effect sizes observed in this survey between both PUs and non-PUs (d = 0.5) and PPUs and non-PPUS (d = 0.7). The results of the ANOVA demonstrated a consistent negative association between pornography use and sexual distress, irrespective of gender. It is possible that sexual discomfort may be associated with increased frequencies of masturbation, pornography use, and extramarital activities among both PUs and PPUs, particularly within a religious context where these behaviors are often considered illegal or condemned. To support this possibility, we observed higher levels of distress in men within the user/non-user groups when the frequency of these sexual behaviors was greater. Similarly, we found increased distress in women within the Pornography Users (PPUs)/Non-PPUs group when the frequency of these sexual behaviors was higher among women. These findings suggest that for addressing PUs’ and PPUs’ concerns, paying attention to cultural and religious beliefs is crucial because distress can be due to fear of disclosure of some forbidden and condemned behaviors such as extramarital and masturbation activities. Another possible explanation is that individuals experiencing higher levels of sexual distress may turn to pornography or masturbation as a coping mechanism.

Some limitations should be considered in the interpretation of the present study’s results. Considering the relatively small sample size, larger surveys are needed to corroborate the present study’s findings. Due to recall bias in self-report studies and the sampling merely from heterosexual married students (due to legal and religious restrictions), generalization of the findings to other populations should be made cautiously. However, this study also had several strengths, such as nationwide sampling on a sensitive topic in a conservative context, online sampling with participant anonymity, use of standardized assessment tools, inclusion of men and women, and a focus on a higher-risk group such as university students.

## Conclusion

Pornography use was reported among 39.6% of students (51.7% of men and 33.6% of women). Problematic pornography use is estimated in 9.5% of overall participants (17.4% of men and 5.6% of women). In Univariate analysis, among all variables, the three largest effect sizes among PUs and non-users were observed for higher sexual distress (d = 0.5), higher masturbation (d = 0.5), and lower religiosity (d = 0.6). Among PPUs and non-PPUs sexual distress (d = 0.7), masturbation (d = 0.6), and sexual desire (d = 0.5) showed the largest effect sizes.

The associated factors with pornography use in logistic regression included being male, lower education, fewer children, higher masturbation, more extramarital relationships, less religiosity, more sexual excitement, and more sexual distress.

A two-way analysis of variance conducted to investigate the effects of gender (male and female) and pornography use (non-user, PUs, and PPU) on the three variables of sexual satisfaction, function, and distress showed that gender or pornography use had no significant effects on sexual satisfaction, women had worse sexual function regardless of pornography use, and pornography users experienced higher sexual distress regardless of gender.

Due to the frequency and factors related to this phenomenon, it is crucial that clinicians provide appropriate pornography literacy programs [60] and introduce reliable sources of sexual information. Differentiation between realistic sexual expectations and the sexual ideals portrayed by the media in the pornography context is important for young people.

## Data Availability

No datasets were generated or analysed during the current study.

## Abbreviations

PU: Pornography use
PUs: Pornography users
non-PPUs: Non-problematic pornography users
PPUs: Problematic pornography users
